# Low Molecular Weight Oligosaccharide from *Panax ginseng* C.A. Meyer against UV-Mediated Apoptosis and Inhibits Tyrosinase Activity In Vitro and In Vivo

**DOI:** 10.1155/2021/8879836

**Published:** 2021-02-26

**Authors:** Yu-Lin Dai, Di Yang, Lai-Hui Song, Hong-Mei Yang, Jiang-Bo Yu, Fei Zheng, Hao Yue, Chang-Bao Chen, En-Peng Wang

**Affiliations:** ^1^Jilin Ginseng Academy, Changchun University of Chinese Medicine, Changchun 130117, China; ^2^Postdoctoral Work Station of Jilin Aodong Medicine Group Co., Ltd., Dunhua 133700, China; ^3^Jilin Provincial Key Laboratory of Ginseng Chemistry and Pharmacology, Changchun 130117, China

## Abstract

To find new anti-UV and whitening agents, 21 fractions isolated from three preparations of ginseng (white, red, and black ginseng) were screened, and their antioxidant effects on AAPH- or H_2_O_2_-induced damage were investigated. Furthermore, the protective effect against UV-mediated apoptosis and the tyrosinase inhibitory activity of the targeted fractions were evaluated in vitro and in a zebrafish model. Among all fractions, F10 from white ginseng was selected as having the strongest anti-UV and antimelanogenesis activities. This fraction exhibited excellent inhibitory effects on the pigmentation of zebrafish, which may be due to its potential tyrosinase inhibitory activity. Additionally, the chemical composition of F10 was evaluated by UPLC-MS and NMR instruments. The results indicated that F10 had a carbohydrate content of more than 76%, and the weight-average molecular weight was approximately 239 Da. Disaccharide sucrose was the main active compound in F10. These results suggest that F10 could be used as an ingredient for whitening cosmetics and regarded as an anti-UV filter in the future.

## 1. Introduction

Human skin, an organ with different types of cells, is mainly influenced by extrinsic factors such as UV radiation, which causes coarse wrinkles, dyspigmentation, and skin laxity [1]. The cosmetic industry is a multibillion dollar-level industry with increasing research every year. Natural products isolated from plant and animal resources are potential active cosmetic candidates for treating various skin disorders and inflammation and protection against UV irradiation [2]. Excessive solar exposure, such as that to UVB radiation, is toxic to the skin, inducing skin aging, DNA skin damage, and tumorigenesis. Several natural active molecules have potential skin care applications due to their different bioactivities [3]. Excess melanin is regarded as hyperpigmentation in the skin. The mechanism of hyperpigmentation involves tyrosinase utilizing L-tyrosine to synthesize L-DOPA, which is then oxidized to L-DOPA quinone. Inhibiting tyrosinase activity is one of the most commonly used strategies in the development of whitening agents [4]. Elevations in inflammatory and oxidative stress are responsible for stimulating melanogenesis. Naturally derived molecules with advantages such as low toxicity and high effectiveness in the treatment of skin lightening are gaining interest in the cosmetic industry [5]. Evidence of natural active molecules inhibiting lipid oxidation was found, exhibiting health-promoting activities such as antiaging, anti-inflammatory, and anticancer effects [6].

*Panax ginseng* C.A. Meyer, known as ginseng, is one of the most famous traditional medicinal herbs in oriental regions, especially in China and Korea [7]. Traditional medicinal theory believes that different ginseng preparations show different activities because the composition has changed. Generally, processed ginseng is mainly divided into white (sun-dried) ginseng [8], red (steamed) ginseng [9], and black (fermented) ginseng [10]. Ginseng presents a number of active compounds, such as polysaccharides, ginsenosides, and peptides [11]. Moreover, previous extensive literature reported the antiaging, wound and ulcer healing, antidiabetic, immunoregulatory, and anticancer activities of ginseng [12]. In recent years, ginseng has become a new candidate in the application of dermatologic therapy and was shown to change signal factors in skin tissue, showing anti-UV, antiwound and injury, antiskin cancer, and antihair loss therapeutic effects [13].

The chemical structure of water-soluble oligosaccharides isolated from ginseng is mainly composed of the *α*-Glcp-(1 ⟶ 6)-*α*-Glcp-(1 ⟶ 4)-*α*-Glcp unit. The oligosaccharides in ginseng were identified as potent B and T cell stimulators [14] and antioxidants [15]. Previous reports found that ginseng-derived low molecular weight oligosaccharides contain 97.48% glucose residues, which could be a potent antitumor immunopotentiator by activating macrophages [16]. Antitumor and immunomodulatory activities of water-soluble oligosaccharides isolated from ginseng were also investigated [17]. To the best of our knowledge, the influence and comparison of different processed ginseng products on the inhibition of melanin and anti-UV effects have not been elucidated thus far. Thus, in this study, we isolated 21 active fractions with different polarities from three processed ginseng products. The anti-UV effect of the fractions in HaCaT cells, as well as the antimelanogenesis activity of the fractions in B16F10 melanoma cells, was evaluated. We also determined the weight-average molecular weights and chemical composition of oligosaccharides from sun-dried ginseng.

## 2. Materials and Methods

### 2.1. Extraction of Active Fractions

**Figure 1:**
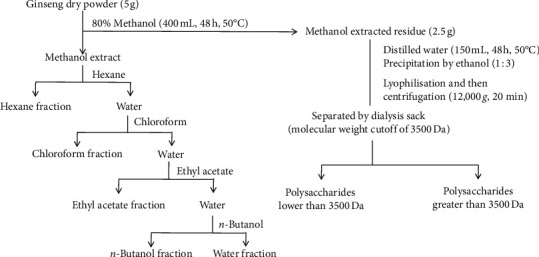
Preparation of 21 fractions from three types of ginseng.

White and red ginseng samples were collected in Fusong County, China, in October 2017 and identified by Professor Shu-Min Wang of Changchun University of Chinese Medicine (Changchun, China). Black ginseng was obtained from our previous study, and the composition of black ginseng was evaluated [10]. The extraction of active fractions was performed in accordance with a previously described protocol [18], as shown in Figure 1. The three types of ginseng were ground into powder separately. Five grams of the powder was extracted in 80% methanol in a waterbath at 50°C for 48 h. The extract solution was concentrated and divided into water and hexane layers and then water and chloroform layers that were further partitioned between water and ethyl acetate. The water layer was subsequently separated with *n*-butanol, and the *n*-butanol fraction was evaporated under vacuum. The residue extracted with methanol was air-dried at 40°C and weighed to approximately 2.5 g. Then, the residue was extracted by distilled water for water-soluble compound extraction. Precipitation from the water extraction was conducted by adding ethanol, and the precipitate was subsequently separated by dialysis depending on the molecular weight. All samples were lyophilized and stored at −20°C for further analysis. Fractions are named as shown in Table 1. The proximate compositions of all ginseng samples were measured following the Association of Official Analytical Chemists methods and a previous study [19, 20].

### 2.2. Cell Culture

Vero cells, HaCaT cells, and B16F10 cells were used for evaluation of the antioxidant, anti-UV, and antimelanogenesis effects in vitro, respectively. All cells were purchased from American Type Culture Collection (ATCC, Manassas, VA, USA). The cells were cultured at 37°C in a 5% CO_2_ incubator and in Dulbecco's modified Eagle's medium maintained with 10% (*v/v*) fetal bovine serum and 1% (*v/v*) penicillin and streptomycin, which were obtained from a commercial source (Gibco Inc., Grand Island, NY, USA).

Cell viability and ROS levels in 2,2′-azobis(2-amidinopropane) dihydrochloride- (AAPH-) or H_2_O_2_-induced Vero cells are treated with active fractions.

In AAPH-induced Vero cells, a colorimetric 3-(4,5-dimethylthiazol-2-yl)-2,5-diphenyltetrazolium bromide (MTT) assay was carried out to detect cytotoxicity. ROS levels were measured by 2′,7′-dichlorofluorescin diacetate (DCFH-DA), which is regarded as an oxidation-sensitive and fluorescence detectable agent [21]. Briefly, Vero cells were seeded at a density of 1 × 10^5^ cells/mL in 96-well culture plates. Different concentrations (12.5, 25, and 50 *μ*g/mL) of 21 active fractions were added to the cells after 24 h of seeding. Following 1 h of incubation with the fractions, the cells were treated with AAPH (10 mM); untreated cells were regarded as the control. The cell viability and ROS production of cells were measured at 24 h after sample treatment. For cell viability detection, the cells were stained with 2 mg/mL MTT solution for 3 h in the dark and then measured at 540 nm wavelength. For ROS measurement, the cells were stained for 10 min with a 5 *μ*g/mL DCFH-DA solution in the dark and then detected by fluorescence spectroscopy (excitation wavelength: 485 nm; emission wavelength: 530 nm) with a microplate reader (BioTech, Winooski, VT, USA). The antioxidant effects of the 21 active fractions in H_2_O_2_-induced Vero cells were determined following the protocol described above, but the treatment of AAPH (10 mM) was replaced with H_2_O_2_ (1 mM).

### 2.3. Cell Viability in Active Fractions Treated in UV-Induced HaCaT Cells

For the activities of natural molecules, antioxidant effects are strongly associated with anti-UV effects [22]. According to the primary results of the antioxidant effect of active fractions, strong activity fractions were selected for further study. The UV-induced HaCaT cell survival rate was measured by the MTT assay. Briefly, HaCaT cells were seeded at a density of 1 × 10^5^ cells/mL in 24-well culture plates. The predetermined concentrations (12.5, 25, and 50 *μ*g/mL) of the 21 active fractions were added to the cells after 24 h of seeding. Following 1 h of incubation with the fractions, the cells were treated with UV energies; the value of UV energy was 20 mJ/cm^2^ from the UV chamber (CL-1000L Ultraviolet Crosslinker, Analytik Jena, USA); untreated cells were considered as the control. The survival rate of cells was measured at 48 h after seeding.

### 2.4. Antimelanogenesis Activities of Active Fractions in *α*-Melanocyte-Stimulating Hormone- (*α*-MSH-) Induced B16F10 Cells

#### 2.4.1. Cell Viability of *α*-MSH-Induced B16F10 Cells

The cell viabilities of B16F10 cells treated with the active fractions were assessed by the MTT assay, which was performed according to the protocol described above.

#### 2.4.2. Measurement of Cellular Melanin Contents

Cellular melanin contents were measured using a previously described method [23]. The cells (2 × 10^4^ cells/mL) were stimulated with *α*-MSH (100 nM) and then incubated with selected active fractions (12.5, 25, and 50 *μ*g/mL) for 72 h; the cells were then washed in ice-cold PBS. Briefly, the cells were incubated at 80°C for 1 h in 1 mL of 1 N NaOH/10% DMSO and then vortexed to solubilize melanin; the absorbance at 450 nm was then measured.

#### 2.4.3. Measurement of Cellular Tyrosinase Activity

Cellular tyrosinase activity was measured according to a previously reported method [24]. Briefly, the cells (2 × 10^4^ cells/mL) were cultured in 24-well plates. Sixteen hours after cell seeding, the cells were stimulated with *α*-MSH (100 nM) and then incubated with selected active fractions (12.5, 25, and 50 *μ*g/mL) for 72 h. The cells were washed with PBS and lysed in PBS containing 1% Triton X-100 by freezing and thawing. The lysates were clarified by centrifugation at 13,000 g for 10 min. After protein quantification and normalization, 90 *µ*l of cell lysate (each sample contained the same amount of protein) was incubated in duplicate with 10 *µ*l of 10 mM L-DOPA at 37°C for 1 h. After incubation, dopachrome was monitored by measuring the absorbance at 405 nm using an ELISA reader. The value of each measurement is expressed as the percentage change from the control.

### 2.5. Pigmentation Generation and Melanin Contents in *α*-MSH-Induced Zebrafish Treated with Active Fractions

Healthy adult zebrafish were purchased from Nanjing Yishulihua Biotechnology Co., Ltd., China, and maintained in 28°C water with a normal light/dark cycle [25]. Embryos were randomly arranged in 24-well plates. Each well contained fifteen embryos with 450 *μ*L of embryonic medium. At 7 hours postfertilization (hpf), cells exposed to 25 *μ*L of arbutin (100 *μ*M) were considered the standard positive control. The control group was treated with 25 *μ*L of PBS. Twenty-five microliters of active fractions at different concentrations were added to the wells from 7 to 72 hpf every 24 h. *α*-MSH (100 nM) was added to all tested groups except the negative control group. At 72 hpf, the pigmentation generation of zebrafish was observed under a microscope (Perkin Elmer LS-5B, Austria).

For the determination of melanin content, the protocol of sample treatment was the same as that of the pigmentation generation assay. At 72 hpf, the zebrafish were collected and centrifuged, and then, the pellet was dissolved in 1 mL of 1 N NaOH at 100°C for 30 min. The mixture was then vigorously vortexed to solubilize melanin. The optical density of the supernatant was measured at 490 nm, and the result was compared with that of the control, which was considered to represent 100%. The melanin content was calibrated with the protein amount.

### 2.6. Measurement of Chemical Composition

#### 2.6.1. Proximate Composition and Molecular Weight Evaluation

The proximate composition of the sample was measured following the Association of Official Analytical Chemists methods and a previous study [19, 20]. The weight-average molecular weights were determined by aqueous-phase gel permeation chromatography (TSK-GEL G2500PWXL, 300 mm × 8 mm; eluent: 0.7% Na_2_SO_4_ solution; 0.5 mL/min) with an RI detector at 35°C [26].

#### 2.6.2. UPLC-MS Analysis

An Ultimate 3000 UPLC system (Thermo Fisher Scientific, USA) coupled with an electrospray ionization triple-quadrupole mass spectrometer (TSQ Endura, Thermo Fisher Scientific, USA) was used in this study. Chromatographic separation was carried out with an XAmide column (150 mm × 2.1 mm i.d., 5 *μ*m) using an UltiMate 3000 HPLC system (Dionex, Sunnyvale, CA, USA). The column was kept at 40°C with a mobile phase of 0.1% formic acid (A) and acetonitrile (B) at a flow rate of 0.2 mL/min. Gradient elution began with 80% B and was then programmed as follows: 75% B from 0 min to 5 min, 60% B from 5 min to 10 min, and 85% B from 10 min to 20 min. MS detection was performed using a triple quadrupole mass spectrometer equipped with an electrospray ion source in positive and negative ionization modes.

The optimized mass spectrometric conditions were as follows: positive mode voltage, 3.5 kV; negative mode voltage, 2.5 kV; sheath gas flow, 35 arb; aux gas flow, 10 arb; ion transfer tube temperature, 325°C; vaporizer temperature, 275°C; and mass scan range, *m*/*z* 50–1000.

The sample spectra were compared with those of standards. Standards of maltotriose, *α*-D-lactose monohydrate, maltose, D-galactose, sucrose, D-(+)-xylose, D-glcNAc, rhamnose, D-fructose, D-(-)-arabinose, D-mannose, and D-(+)-glucose were purchased from YuanYe Biotechnology Company (Shanghai, China).

#### 2.6.3. NMR Analysis

NMR spectra of the fractions were measured using a Bruker AV600 600 MHz spectrometer (Bruker Co., Billerica, MA, USA) at 298 K. The fractions were dissolved in D_2_O for NMR experiments [27].

### 2.7. Statistical Analysis

All assays were performed in triplicate and as independent experiments. Values are expressed as the mean ± SE. One-way ANOVA was used to analyze the mean values in GraphPad Prism 5. Student's *t*-test (^*∗*^*p* < 0.05 and ^*∗∗*^*p* < 0.01) was used to analyze the means of the parameters in terms of significant differences.

## 3. Results and Discussion

### 3.1. Screening of Cytotoxicity and ROS Levels in Vero Cells

**Figure 2:**
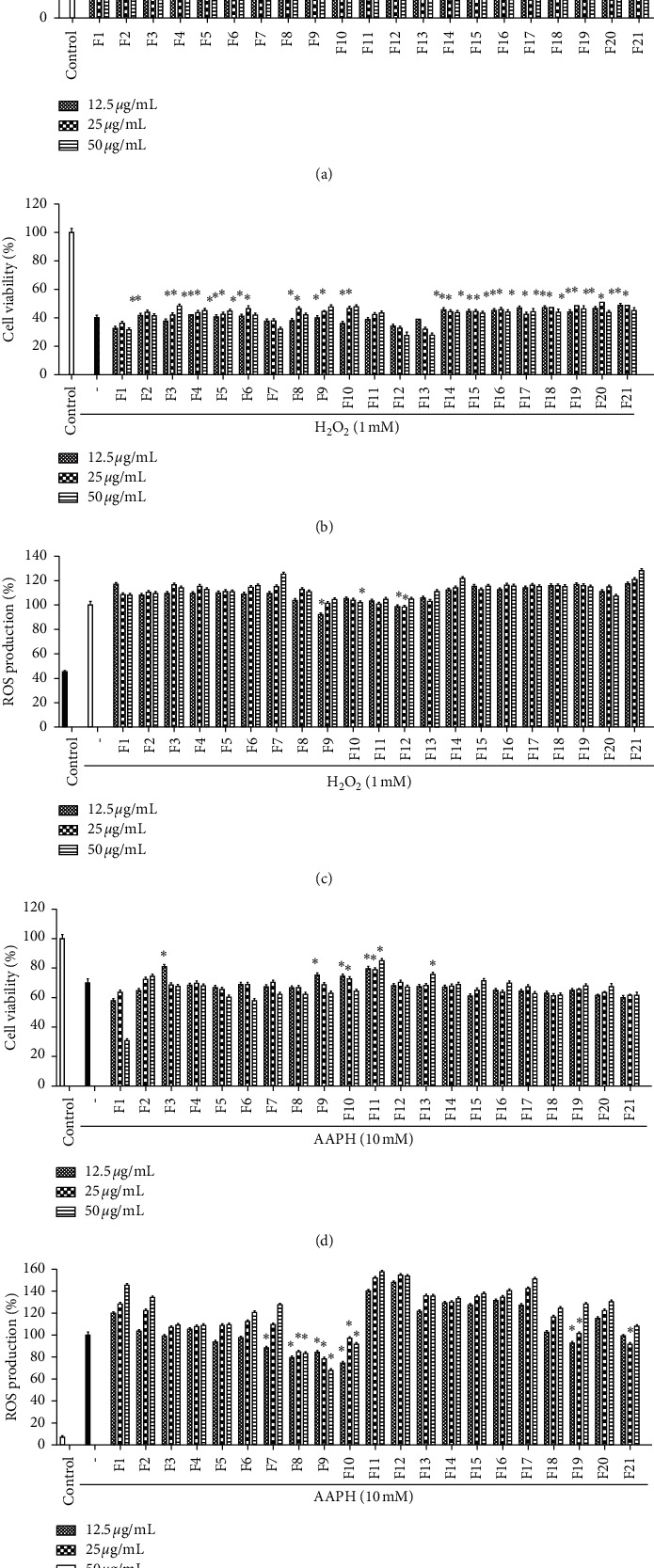
Evaluation of 21 fractions for toxicity and intracellular ROS scavenging activities against oxidative stress on Vero cells. (a) Toxicity assessment of the samples; (b) protective activities of fractions against H_2_O_2_-induced cytotoxicity; (c) scavenging activities of fractions on H_2_O_2_-induced intracellular ROS; (d) protective activities of fractions against AAPH-induced cytotoxicity; (e) scavenging activities of fractions on AAPH-induced intracellular ROS. Results represent the percentage (%) of cell viability and intracellular ROS levels. Experiments were performed in triplicate, and the data are expressed as the mean ± SE. ^*∗*^*p* < 0.05; ^*∗∗*^*p* < 0.001.

Figure 2(a) shows the cytotoxic effect of the extracts from three types of ginseng on Vero cells. The survival rates of cells exposed to the 21 fractions in a concentration-dependent manner ranged from 12.5 to 50 *μ*g/mL. Among the results of the cytotoxicity screening, all fractions had survival rates above 80% at each concentration. Hence, the indicated concentrations were selected for further investigation.

The antioxidant effects of the 21 fractions on ROS production in H_2_O_2_-induced Vero cells were observed using the DCFH-DA assay. The results showed that the protective effect and ROS production were strongly suppressed in Vero cells treated with F9, F10, F11, F12, and F13 (Figures 2(b) and 2(c)). The fluorescence in the control group was low in intensity; whereas, H_2_O_2_ increased the fluorescence, demonstrating that ROS was produced in H_2_O_2_-stimulated Vero cells. Additionally, a large decrease in ROS levels was observed when F9, F10, and F11 were used to pretreat AAPH-induced Vero cells (Figure 2(d)). In AAPH-induced Vero cells, we found that fractions F8, F9, and F10 reduced ROS levels and showed potential protective effects (Figure 2(e)).

### 3.2. Anti-UV Effect in HaCaT Cells

**Figure 3:**
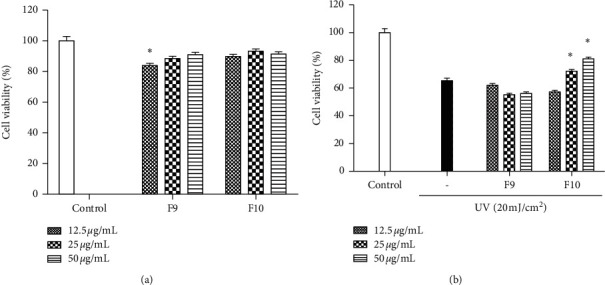
Anti-UV effects of fractions in UV-induced HaCaT cells. (a) Toxicity assessment of the fractions; (b) protective activity of target fractions against UV-induced cytotoxicity. Results represent the percentage (%) of cell viability. Experiments were performed in triplicate, and the data are expressed as the mean ± SE. ^*∗*^*p* < 0.05; ^*∗∗*^*p* < 0.001.

Regarding the antioxidant effect screening of fractions, F9 and F10 were selected for further study. Initially, the cytotoxic effect of the fractions and their protective effects against UV-stimulated cellular damage were evaluated by MTT assay. The cell survival rates of the samples were greater than 80% at all concentrations. This result implies that no cytotoxic effects were found in HaCaT cells (Figure 3(a)). When HaCaT cells were exposed to UV, the cell survival rate decreased to 62%. However, both F9 and F10 increased cell viability at relatively high concentrations, and the two fractions showed high cell viability and significant differences at all concentrations (Figure 3(b)).

### 3.3. Effects of Melanogenic Inhibitors on Tyrosinase Activity in B16F10 Cells

**Figure 4:**
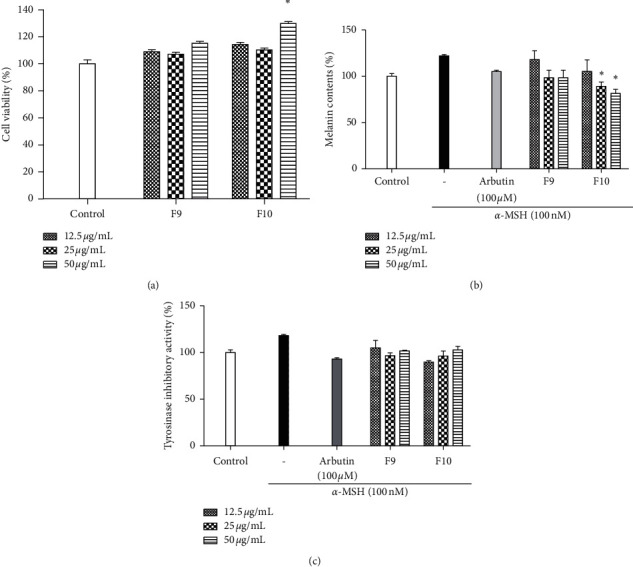
Antimelanogenesis activities of fractions in *α*-MSH-induced B16F10 cells. (a) Toxicity assessment of the fractions; effects of melanogenic inhibitors on (b) melanin synthesis and (c) tyrosinase activity in *α*-MSH-induced B16F10 cells. Arbutin (100 *μ*M) was utilized as positive controls. Experiments were performed in triplicate, and the data are expressed as the mean ± SE. ^*∗*^*p* < 0.05, ^*∗∗*^*p* < 0.001.

To estimate the inhibitory activities, the tyrosinase activity and total melanin content were measured using B16F10 cells. Initially, the results showed that the cell survival rates of F9 and F10 were higher than 90%, indicating low cytotoxicity at different concentrations (Figure 4(a)). We noted substantial reductions in tyrosinase activity and total melanin contents after treatment with F9 and F10 (Figures 4(b) and 4(c)). Arbutin was considered as a positive control and reduced both the tyrosinase activity and total melanin content to a marked degree. For the tested fractions, F9 inhibited both the tyrosinase activity and total melanin content; furthermore, the decreasing rate of these factors in F10-treated cells was greater than that in F9-treated cells (Figures 4(b) and 4(c)).

### 3.4. Effects of Melanin Synthesis in Zebrafish Embryos

**Figure 5:**
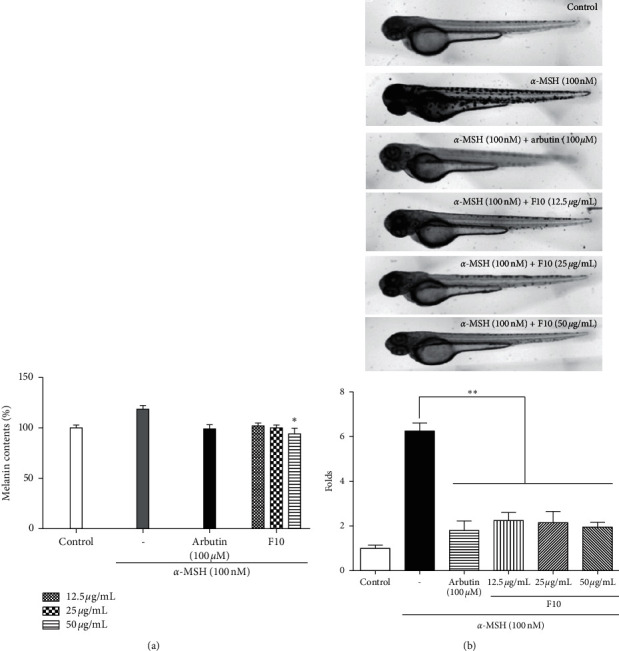
Effects of melanogenic inhibitors on (a) pigmentation and (b) morphological characteristics in zebrafish embryos. The effects on the pigmentations of zebrafish embryos were observed by microscopy. Intensity of embryos was calculated using Image J software.

Zebrafish are used as an animal model for in vivo whitening studies because their genetic makeup is more than 90% similar to that in mammals [25]. Among the 21 fractions exhibiting antioxidant, anti-UV, and antimelanogenesis activities, F10 showed the strongest activities and lowest cytotoxicity in vitro. In Figure 5(a), different concentrations of F10 showed lower melanin contents than those of the *α*-MSH-treated group. The total melanin content of the arbutin treatment group was observed to be reduced in *α*-MSH-induced embryos compared to that of the control.

Figure 5(b) shows the morphological findings of this study. Arbutin, a positive control, exhibited a remarkable inhibition of trunk and yolk sac pigmentation. When the embryos were treated with varying concentrations of F10, the melanin content decreased on the surface of the trunk in a dose-dependent manner. In particular, yolk sac pigmentation was inhibited dramatically after treatment with F10.

### 3.5. Chemical Composition of Each Fraction

**Figure 6:**
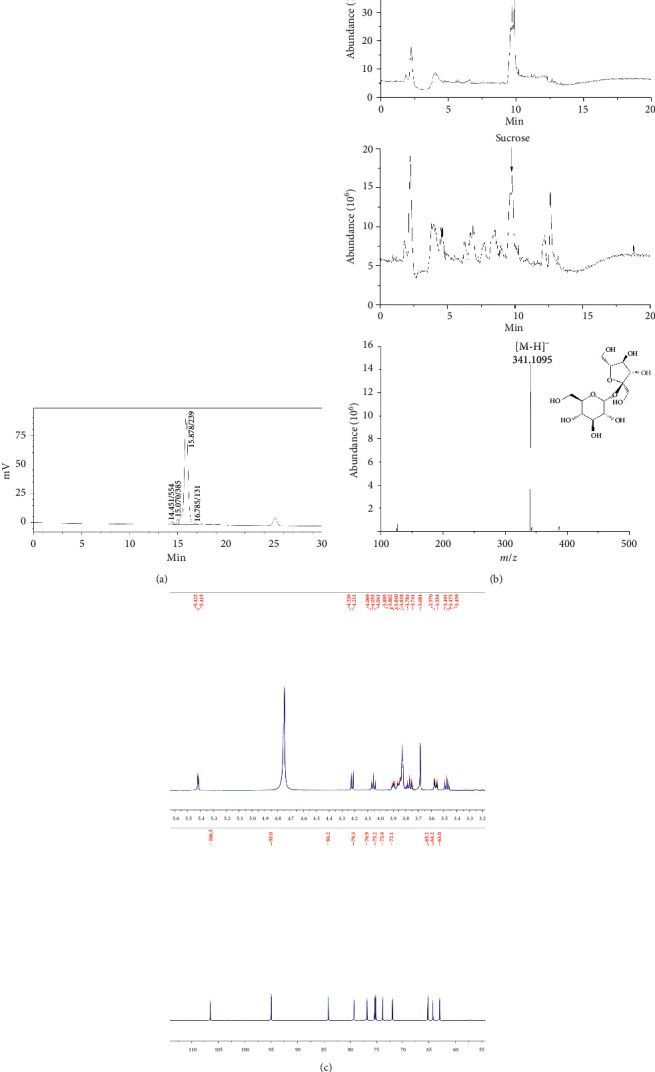
Spectroscopic analysis of F10 from white ginseng. (a) Spectra of weight-average molecular weight; (b) LC-MS spectra; (c) NMR spectra.

F10 is an active pale yellow amorphous powder, soluble in water. The yield of F10 is 28.63%. The chemical composition of F10 is shown in Table 2. Carbohydrates were the main components of F10, constituting approximately 80% of the fraction. The ash, protein, and uronic acid contents of F10 were hardly detected. This result was in accordance with the findings of the previous report [28]. The weight-average molecular weight of F10 was 239 Da (Figure 6(a)). UPLC-MS analysis of the monosaccharide composition of F10 indicated that the content of sucrose was more than 40% based on chromatographic peak area calculations. The mass spectrum of sucrose in F10 is shown in Figure 6(b). In Figure 6(c), ^1^H NMR and ^13^C analysis of F10 indicated peaks characteristic of carbohydrates, as reported previously [29]. The observed peaks agreed well with the reported characteristic peaks of sucrose. Therefore, F10 was regarded as a fraction rich in low molecular weight carbohydrate polymers with strong antioxidant, anti-UV, and antimelanogenesis activities.

Ginseng is a famous herb that has been used for the treatment of spiritlessness and fatigue for thousands of years in China and Korea [30]. Its constituents consist of diverse secondary metabolites, including ginsenosides, polysaccharides, oligosaccharides, phenolic compounds, alkaloids, peptides, and vitamins, which are considered to be the main molecules with various beneficial effects [31]. Recently, there have been new aspects of the therapeutic activity of ginseng used in dermatologic disorders, such as photoaging, wounding, dermatitis, alopecia, and cold hypersensitivity [13]. UV radiation is considered a complete carcinogen due to its tumor-promoting properties. Limited UV exposure mediates the natural synthesis of vitamin D and endorphins in the skin; however, excessive UV exposure poses profoundly high risks, including atrophy, pigmentary changes, wrinkling, and malignancy [32]. Additionally, modern formulations of traditional herbs exist in various forms for the development of cosmetics [33]. It has been reported that red ginseng extracts prevent UV-induced intracellular reactive oxygen species [34] and protect UV-induced skin by regulating the expression of SPT protein [35]. Previous studies have indicated that antioxidant effects are strongly associated with anti-UV effects. In our study, we isolated more than twenty fractions from three types of ginseng to screen potential cosmetic candidates. Interestingly, F10, mainly containing low molecular weight oligosaccharides, was selected as the target active fraction. To the best of our knowledge, this is the first report on the anti-UV effect of oligosaccharides isolated from ginseng.

In this work, F10 was considered to be a water-soluble oligosaccharide-rich fraction mainly composed of sucrose and having a weight-average molecular weight of approximately 239 Da and was obtained from white ginseng. This fraction contained attractive bioactive compounds with various activities. The ratio of active compounds in the contained oligosaccharides may play a key role in the bioactive properties of F10, and the synergistic biological properties were influenced by the composition of F10. There are a number of studies on ginseng polysaccharide; whereas, there are fewer than ten articles on ginseng oligosaccharides. Evidence of the antioxidant effects of ginseng oligosaccharides has been found. Hence, the potential of F10 to have antimelanogenic properties was highly expected.

Ginseng has been proven to have a whitening effect. The inhibitory effect on melanin biosynthesis of phenolic compounds isolated from white and red ginseng was investigated [36]. Minor ginsenosides exerted antimelanogenic effects [37]. For instance, ginsenoside Rh4 reduced the cAMP level and cAMP response-element binding protein level in B16 melanoma cells [38]. However, a previous report showed that ginsenosides Rb1 and Rg1 induce melanogenesis via regulated expression of MITF, tyrosinase, and p-CREB in human skin cells [39]. Hence, the antimelanogenic effects of ginseng remain unclear. F10 isolated from white ginseng is rich in sucrose, which is popular as a cosmetic material [40, 41]. It has been shown that sucrose mixed with other components enhances nasal and ocular peptide absorption [42]. It is clear that the sucrose in F10 synergistically worked with other unknown active compounds. Further studies in the elucidation of additional active compounds in F10 are the emphasis of our work.

The in vitro results acquired in this work showed the successful application of ginseng extract fractions to reduce pigmentation. However, the evidence from the in vivo studies needs to be further investigated. Generally, zebrafish are widely recognized as animal models in drug discovery and toxicological studies. The melanin pigmentation of the zebrafish epidermis allows for the observation of changes without other complicated instruments [43]. There are three types of pigment cells in zebrafish skin: melanophores, xanthophores, and iridophores [44]. Among them, melanophores mainly contribute to the formation of the characteristic longitudinal dark stripes on the surface of zebrafish [45]. Arbutin has strong and stable antimelanogenesis activity in a zebrafish model and was employed as the positive control in the relative bioassay. In this study, the effects of melanogenic inhibition of F10 were evaluated in a zebrafish model. Interestingly, arbutin treatment exhibited inhibitory effects on zebrafish pigmentation. F10 exerted profound inhibitory effects on zebrafish pigmentation, most likely as a consequence of its tyrosinase activity-inhibitory potential over the same period. Additionally, F10 has been shown in previous results to possess excellent antioxidant activities and high oligosaccharide content, thus making it a potential candidate for cosmetic applications. In summary, F10 was evaluated in regard to its potential efficacy as an anti-UV and skin-whitening agent in both in vitro and zebrafish models. These results support our hypothesis that F10 is likely to be useful to the cosmetic and medicinal industries.

## 4. Conclusions

In this study, among the 21 active fractions, F10 from ginseng was evaluated as a naturally derived skin-whitening agent and also exhibited anti-UV effects in both cellular experiments and zebrafish models. Furthermore, evidence of significant inhibitory effects against tyrosinase and melanin synthesis of F10 was found. Hence, F10, containing low molecular weight oligosaccharides, from ginseng can be developed as a potential cosmetic and medicinal material.

## Figures and Tables

**Table 1:** The list of fractions from three types of ginseng and each yield.

Fractions	Red ginseng	White ginseng	Black ginseng
Hexane	F1 (5.8 mg)^*∗*^	F6 (23.8 mg)	F11 (7.2 mg)
Chloroform	F2 (9.1 mg)	F7 (21.5 mg)	F12 (57.5 mg)
Ethyl acetate	F3 (28.3 mg)	F8 (14.5 mg)	F13 (33.9 mg)
Butanol	F4 (235.9 mg)	F9 (315 mg)	F14 (230.9 mg)
Water	F5 (1404.8 mg)	F10 (1431.5 mg)	F15 (1822.1 mg)
Polysaccharides (>3500 Da)	F16 (135 mg)	F17 (60 mg)	F18 (220 mg)
Polysaccharides (<3500 Da)	F19 (4.7 mg)	F20 (16.8 mg)	F21 (4.2 mg)

^*∗*^For all, F + Arabic numbers such as “F1” represents the fraction name; the value in parentheses indicates the yield of the fraction.

**Table 2:** Chemical compositions of F10.

State	F10
Carbohydrate (%)	76.3 ± 4.2%
Protein (%)	Trace
Uronic acid (%)	Trace
Ash	0.3%
Weight-average molecular weight (Da)	239

## Data Availability

The data used to support the findings of this study are available from the corresponding author upon request.

## Supplementary Materials

Supplementary Materials Figure S1. Two-dimensional nuclear magnetic resonance (COSY) imaging of F10 from white ginseng. Figure S2. Two-dimensional nuclear magnetic resonance (HSQC) imaging of F10 from white ginseng.
