# Supplementary material for: Low Molecular Weight Oligosaccharide from Panax ginseng C.A. Meyer against UV-Mediated Apoptosis and Inhibits Tyrosinase Activity In Vitro and In Vivo
Source: Evid Based Complement Alternat Med. 2021 Feb 26;2021:8879836. doi: 10.1155/2021/8879836 (PMC7935584; doi:10.1155/2021/8879836)
Supplement: Supplementary Materials — Figure S1. Two-dimensional nuclear magnetic resonance (COSY) imaging of F10 from white ginseng. Figure S2. Two-dimensional nuclear magnetic resonance (HSQC) imaging of F10 from white ginseng. [file 8879836.f1.docx]

**Supplementary File**

**Figure S1**: Two–Dimensional Nuclear Magnetic Resonance (COSY) imaging of F10 from white ginseng.

**Figure S2**. Two–Dimensional Nuclear Magnetic Resonance (HSQC) imaging of F10 from white ginseng.
